# 10-year follow-up results of the prospective, double-blinded, randomized, controlled study on autologous bone marrow buffy coat grafting combined with core decompression in patients with avascular necrosis of the femoral head

**DOI:** 10.1186/s13287-020-01810-8

**Published:** 2020-07-16

**Authors:** Mengyuan Li, Yuanchen Ma, Guangtao Fu, Ruiying Zhang, Qingtian Li, Zhantao Deng, Minghao Zheng, Qiujian Zheng

**Affiliations:** 1 Division of Joint Osteopathy and Traumatology, Center of Orthopedics Surgery, Guangdong Provincial People’s Hospital, Guangdong Academy of Medical Sciences, School of Medicine, South China University of Technology, 106 Zhongshan 2nd Road, Yuexiu District, Guangzhou, 510080 People’s Republic of China; 2 Centre for Orthopaedic Translational Research, School of Surgery, The University of Western Australia, M Block, QE2 Medical Centre, Monash Ave., Nedlands, WA 6009 Australia

**Keywords:** Avascular necrosis of the femoral head, Core decompression, Autologous bone graft, Bone marrow buffy coat

## Abstract

**Background:**

Avascular necrosis of the femoral head (ANFH) is a severely disabling disease of the hip. Several clinical trials have shown promising outcomes on the use of mesenchymal stem cells for the treatment of ANFH, but long-term clinical assessments are lacking. Previously, we reported the 2-year follow-up results of a prospective, double-blinded, randomized, controlled study on autologous bone marrow buffy coat grafting combined with core decompression in patients with ANFH. Here, we report the 10-year follow-up results of this study.

**Methods:**

We recruited 43 (53 hips) patients from 2009 to 2010. The hips were randomly allocated to code decompression (CD) with or without bone marrow buffy coat (BBC) grafting. Participants underwent follow-up at 24, 60, and 120 months postoperatively. The visual analogue scale (VAS), Lequesne algofunctional index, and Western Ontario and McMaster Universities Arthritis Index (WOMAC) osteoarthritis scores were recorded. Survival rate analysis and prognostic factor analysis were performed. The endpoint was defined as progression to Ficat stage IV or conversion to hip arthroplasty.

**Results:**

A total of 31 patients (41 hips) were included in the final analysis. The CD + BBC group had better subjective assessment scores than the CD group. The average survival times were 102.3 months and 78.1 months in the CD + BBC group and CD group, respectively (log-rank test, *P* = 0.029). In the univariate Cox proportional hazards regression model, age [hazard ratio (HR) = 1.079, *P* = 0.047] and preoperative Ficat stage (HR = 3.283, *P* = 0.028) indicated a high risk for progression, while the use of BBC (HR = 0.332, *P* = 0.042) indicated a low risk. Preoperative Ficat stage III was isolated as an independent risk factor for clinical failure in the multivariate model (HR = 3.743, *P* = 0.018).

**Conclusion:**

The 10-year follow-up results of this prospective, double-blinded, randomized, controlled study showed that the use of autologous BBC in combination with core decompression was more effective than the use of core decompression alone.

**Trial registration:**

ClinicalTrials.gov, NCT01613612. Registered on 13 December 2011—retrospectively registered

## Background

The aetiology of avascular necrosis of the femoral head (ANFH) is multi-factorial, including trauma, corticosteroid use, and excessive alcohol consumption [1]. ANFH is often initiated by impaired circulation of the femoral head, leading to ischaemia due to increased intraosseous pressure and consequently causing osteonecrosis of the subchondral plate and osteoarthritis. Because ANFH is progressive and eventually leads to hip dysfunction and disability, total hip arthroplasty (THA) is the ultimate treatment for terminal ANFH. Nonetheless, despite the success of primary THA, recent registry data have revealed that the revision burden is 4.7~13.8%, which suggests poor prosthetic durability [2]. Therefore, concerns are growing about hip joint preservation prior to subchondral bone collapse, especially in younger and physically demanding individuals. Conservative procedures, in terms of core decompression and vascularized or avascular bone transplantation, have been widely used for early-stage ANFH to delay pathological progression, and several clinical studies have shown favourable short-term and mid-term results [1]. Core decompression, which can directly mitigate intraosseous hypertension, is the most common treatment because of its advantage of minimal invasion. However, the efficacy of traditional core decompression is inconsistent. Some short-term studies have indicated that the clinical failure rate reaches 20~70%, even for those in the early stage (Ficat stage I/II). Bone grafting has been employed to provide subchondral structural support to allow healing and remodelling [3]. Based on the current literature, bone grafting can postpone femoral head collapse and preserve hip joint function. However, this procedure is more invasive and can lead to donor site morbidity and nerve palsy.

In the last decade, accumulating evidence has indicated that a decreased pool of osteoprogenitor cells in the bone marrow of the femoral head is associated with the aetiology of ANFH. Thus, there has been enthusiasm for applying osteogenic precursors to necrotic lesions in ANFH [4]. With the use of cell therapy, such as mesenchymal stem cell (MSC) transplantation or bone marrow aspirate concentrate injection, previously published reports have demonstrated benefits, including significant pain relief, reduced time to collapse, decreased lesion sizes, and functional restoration [5–7]. Although some of the studies had high evidence levels, the follow-up times were relatively short [3, 8–13]. There is also a lack of long-term data evaluating the efficacy. Previously, we reported the outcome of a 2-year study showing that implantation of the autologous bone marrow aspirate bone marrow buffy coat (BBC) combined with core decompression is promising for pain relief and postponement of THA [3]. In the present study, we continued the follow-up of this prospective, randomized, controlled trial to further investigate the longer-term outcomes of the study. We report the 10-year outcome, showing that the use of autologous BBC in combination with core decompression is more effective than the use of core decompression alone.

## Methods

### Study design

This study design was approved by the Institutional Review Board of Guangdong Provincial People’s Hospital and was performed in strict accordance with the ethical standards stipulated in the 1964 Declaration of Helsinki and its later amendments. Signed informed consent for participation was obtained from all study patients. The trial protocol was submitted to ClinicalTrials.gov, and the trial registration number is NCT01613612.

Based on the results of Gangi et al. [14], we determined that at least 20 hips per group were necessary to detect a difference by using the Wilcoxon-Mann-Whitney test with a bilateral *α* of 0.017 and a power of 80%. We included a minimum of 26 hips in each group to compensate for the patients lost to follow-up.

### Patients

We recruited eligible patients from those admitted to the Center of Orthopedic Surgery from June 2009 to October 2010. Plain radiographs of the bilateral hips in the anteroposterior and frog-leg lateral positions as well as magnetic resonance imaging (MRI) were performed in all patients. We confirmed the diagnosis of ANFH based on the clinical history and the radiographic lesions in the femoral head.

The inclusion criteria were as follows: the subjects (1) were 18 to 55 years old; (2) had notable hip pain; (3) had normal, minor, or mixed osteopenia or had crescent signs detected on plain radiographs; and (4) stopped steroid treatment for at least 6 months.

The exclusion criteria were as follows: (1) < 18 or > 55 years old; (2) terminal stage of ANFH with the presence of secondary osteoarthritic changes such as osteophyte formation, narrowed joint gap, and osteosclerosis; (3) history of fracture in the proximal femur, tumours, or any other concomitant lower extremity diseases; (4) previous history of any surgical treatment in terms of core decompression, bone grafting, titanium implantation, or osteotomy; (5) previous history of any conservative treatment, such as extracorporeal shock wave therapy, hyperbaric oxygen, and alendronate; (6) inflammatory arthritis, including rheumatoid arthritis, suppurative arthritis, and gouty arthritis; (7) having received steroid treatment in the last 6 months; and (8) pregnancy.

After meeting the inclusion criteria, the participants provided informed consent, and their baseline characteristics were assessed. The hips were randomly allocated to receive core decompression (CD) + an autologous bone graft (BG) or core decompression + an autologous bone graft + BBC (CD + BG + BBC) by a randomization schedule, which was generated by computer-based block randomization.

### Surgical technique

All surgical procedures were performed under continuous epidural anaesthesia. For core decompression of the femoral head, we first determined the optimal entrance point for drilling, and then a 1.5-cm incision was made at the level of the greater trochanter. A 3.0-mm-diameter Kirschner wire (K-wire) was introduced into the necrotic area with the tip placed in the subchondral bone area approximately 2 to 3 mm from the articular cartilage. Next, a 10-mm-diameter trephine was drilled through the K-wire to the necrotic region. A cylinder of bone from the femoral neck and head was obtained. The necrotic proximal part was eliminated, and the healthy part was used for BBC grafting. The necrotic tissue remaining in the femoral head was removed with the bone curette. All the above steps were performed under C-arm X-ray guidance.

For bone marrow collection, a 50-mL syringe, heparinized in advance, was used to harvest the bone marrow from the posterior superior iliac spine. The bone marrow was centrifuged at 1500 rpm for 10 min in a bench-top centrifuge (Eppendorf, AG 22331, Hamburg, Germany) with a sterilized chamber. The bone marrow was separated into three phases after centrifugation. We collected a total of 1 mL of bone marrow concentrate from the interface containing enriched bone marrow cells with a sterilized transfer pipette, and then, the bone marrow concentrate was seeded on the cylindrical bone drop by drop to allow the cells to anchor onto the bone surface. A total of 10 μL of bone marrow concentrate was kept for cell counting after the surgery. The average number of bone marrow cells loaded onto the cylindrical bone was approximately 3 × 10^9^ nucleated cells.

The bone graft with or without BBC was inserted and impacted into the necrotic region through the CD track with the guidance of C-arm X-ray. After surgery, the patients were instructed to remain non-weight-bearing for 4 weeks. Surgical complications were monitored after the operation.

### Outcome assessment

Preoperatively, blinded evaluators collected baseline demographic information, including age, sex, aetiological factors, presurgical Ficat stage of ANFH, and location of the defect. Plain X-ray radiography and MRI were used to determine the Ficat stage of ANFH and the location of the necrotic lesions. All participants received follow-up at 24, 60, and 120 months postoperatively. Anteroposterior and frog-leg lateral radiographs were taken during each clinical assessment. The radiographic progression of ANFH was determined based on the Ficat classification system. The primary outcomes included visual analogue scale (VAS), Lequesne algofunctional index, and Western Ontario and McMaster Universities Arthritis Index (WOMAC) osteoarthritis scores. The secondary outcome was the clinical failure rate of the operated hips at 5 years and at the time of the final follow-up. The clinical failure rate was defined as the proportion of hips that progressed to Ficat stage IV or that were subjected to THA.

### Statistical analysis

The SPSS 22.0 software package (IBM Inc., USA) was used for statistical analysis. The data were described as median, maximum, and minimum values. The chi-square test and Fisher’s exact test were used for categorical variables. The statistical significance of the demographic data and functional assessments between the two groups were determined by the Wilcoxon-Mann-Whitney test. Clinical survival was compared between each group with Kaplan-Meier survivorship analysis, and statistical significance was determined by the log-rank test. A Cox proportional hazards regression model was employed to detect the risk factors affecting the survival of the femoral head. The level of statistical significance for all tests was defined at *P* <  0.05.

## Results

### Demographic and baseline characteristics

**Fig. 1:**
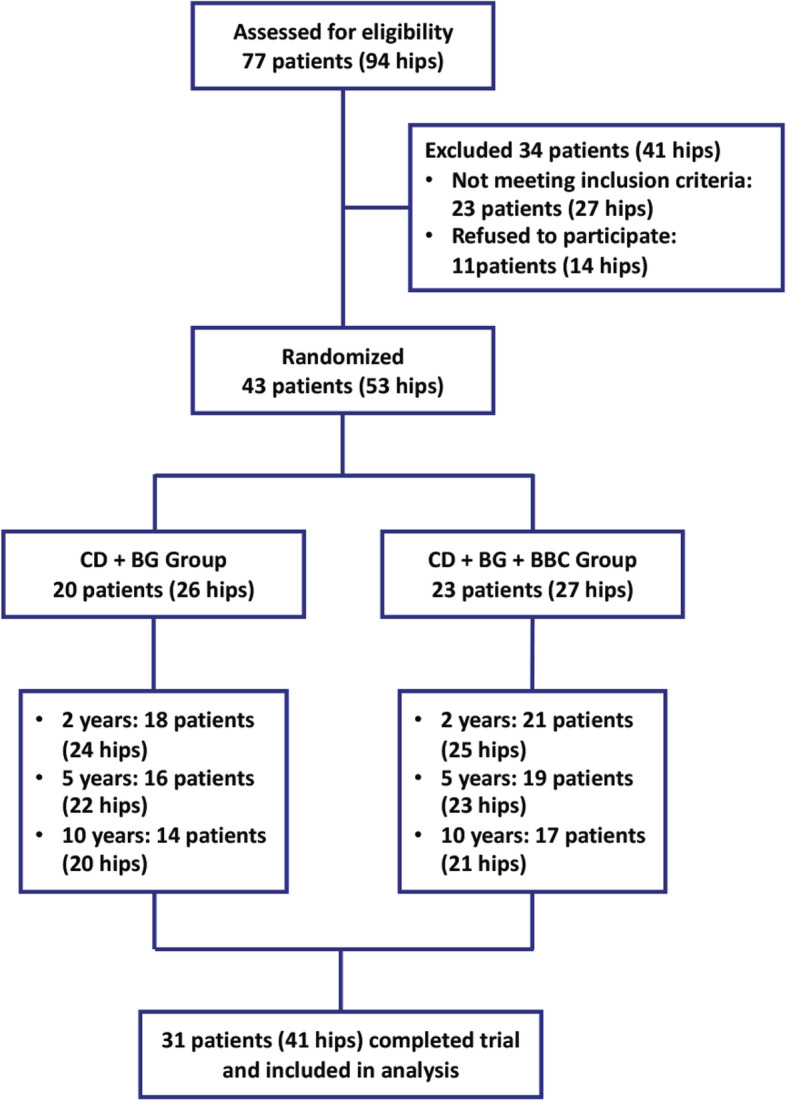
Flowchart of the present study. CD core decompression, BG bone grafting, BBC bone marrow buffy coat

A total of 77 patients (94 hips) were screened for eligibility. After excluding 34 patients (41 hips), 43 (53 hips) patients who met the inclusion criteria were recruited and randomly allocated into the CD + BG or CD + BG + BBC group. A total of 12 patients (12 hips) were lost to follow-up. In the CD + BG group, 3 patients lost contact, and 3 patients quit this trial for personal reasons. In the CD + BG + BBC group, 2 patients lost contact, and 4 patients left the study for personal reasons. A total of 31 patients (41 hips) were included in the final analysis (Fig. 1). Table 1 shows the demographic data and clinical baseline characteristics of the included subjects. The data of the two groups were homogeneous.

**Table 1:** Demographic and baseline data of included patients

	CD + BC group (*n* = 14)	CD + BC + BBC group (*n* = 17)	*P* value
Number of hips	20	21	
Age, years	38.2 ± 8.1	34.1 ± 8.0	0.112
Sex			1.000
Male	10	12	
Female	4	5	
Aetiology			0.900
Steroid	9	10	
Alcohol	5	6	
Idiopathic	6	5	
Ficat stage			0.867
II	11	11	
III	9	10	
VAS	4.5 (2 to 10)	4 (2 to 10)	0.703
Lequesne index	10 (3 to 20)	9 (1 to 21)	0.539
WOMAC	33 (8 to 91)	21 (2 to 80)	0.179

*CD* core decompression, *BG* bone grafting, *BBC* bone marrow buffy coat, *VAS* visual analogue scale, *WOMAC* Western Ontario and McMaster Universities Arthritis Index osteoarthritis scoring

### Functional outcome changes during follow-up

**Fig. 2:**
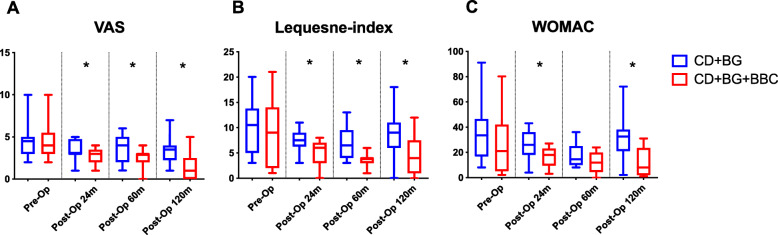
Functional outcome before and after surgery. Error bars denote the minimum and maximum value of each group. Segments with significant statistical differences (*P < 0.05) between the groups were marked with asterisks. CD core decompression, BG bone grafting, BBC bone marrow buffy coat, VAS visual analogue scale, WOMAC Western Ontario and McMaster Universities Arthritis Index osteoarthritis scoring

The VAS score was significantly lower in the CD + BG + BBC group at each postoperative follow-up time point than in the CD + BG group (24 months: *P* = 0.028; 60 months: *P* = 0.018; 120 months: *P* = 0.001, respectively). In terms of the Lequesne index, the CD + BG + BBC group scored significantly better 24 months (*P* = 0.005), 60 months (*P* = 0.001), and 120 months (*P* = 0.001) postoperatively than the CD + BG group. The WOMAC score was significantly lower in the CD + BG + BBC group than in the CD + BG group 24 months (*P* = 0.010) and 120 months (*P* <  0.001) after surgery (Table 2; Fig. 2). Furthermore, 4 patients in the CD + BG group underwent THA, while 2 patients in the CD + BG + BBC group underwent THA (Fisher’s exact *P* = 0.833, Table 3).

**Table 2:** Functional outcomes [mean (min to max)] before and after surgery

		Pre-operation	24 months post-operation	60 months post-operation	120 months post-operation
VAS	CD + BC	4.5 (2 to 10)	3 (1 to 5)	4 (1 to 6)	3.5 (1 to 7)
	CD + BC +BBC	4 (2 to 10)	3 (1 to 4)	3 (0 to 4)	1 (0 to 5)
*P* value		0.703	0.028	0.018	0.001
Lequesne index	CD + BC	10 (3 to 20)	7.5 (3 to 11)	6.5 (3 to 13)	9 (0 to 18)
	CD + BC +BBC	9 (1 to 21)	6 (0 to 8)	4 (1 to 6)	4 (0 to 12)
*P* value		0.539	0.005	0.001	0.001
WOMAC	CD + BC	33 (8 to 91)	26 (4 to 43)	14.5 (8 to 36)	32.5 (2 to 72)
	CD + BC +BBC	21 (2 to 80)	18 (3 to 27)	12 (0 to 24)	8 (1 to 31)
*P* value		0.179	0.010	0.131	< 0.001

*CD* core decompression, *BG* bone grafting, *BBC* bone marrow buffy coat, *VAS* visual analogue scale, *WOMAC* Western Ontario and McMaster Universities Arthritis Index osteoarthritis scoring

**Table 3:** Number of patients receiving THA during the follow-up

Group	Outcome	Number
CD + BG	Hip preserved	16
	THA	4
CD + BG + BBC	Hip preserved	19
	THA	2

*THA* total hip arthroplasty, *CD* core decompression, *BG* bone grafting, *BBC* bone marrow buffy coat

### Survivorship during follow-up

**Fig. 3:**
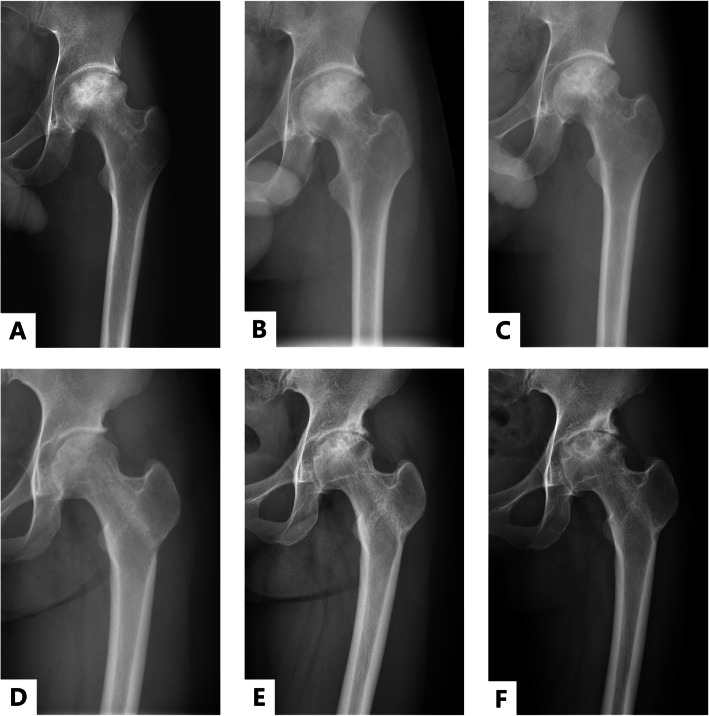
Anteroposterior plain sequence from the representative cases. **a**, **b** and **c** show the 2-year, 5-year, and 10-year postoperative anteroposterior plain of a 24-year-old male with the diagnosis of ANFH (Ficat stage IIB) caused by steroid. He was assigned in the CD + BG + BBC group. At 10-year follow-up, the necrotic region progressed slightly. **d**, **e**, and **f** show the 2-year, 5-year, and 10-year postoperative anteroposterior plain of a 26-year-old female, who underwent the treatment of CD + BG because of idiopathic ANFH (Ficat stage IIA). At 10-year follow-up, it showed progressive decrease in the necrotic region and the hip showed significant degeneration

**Fig. 4:**
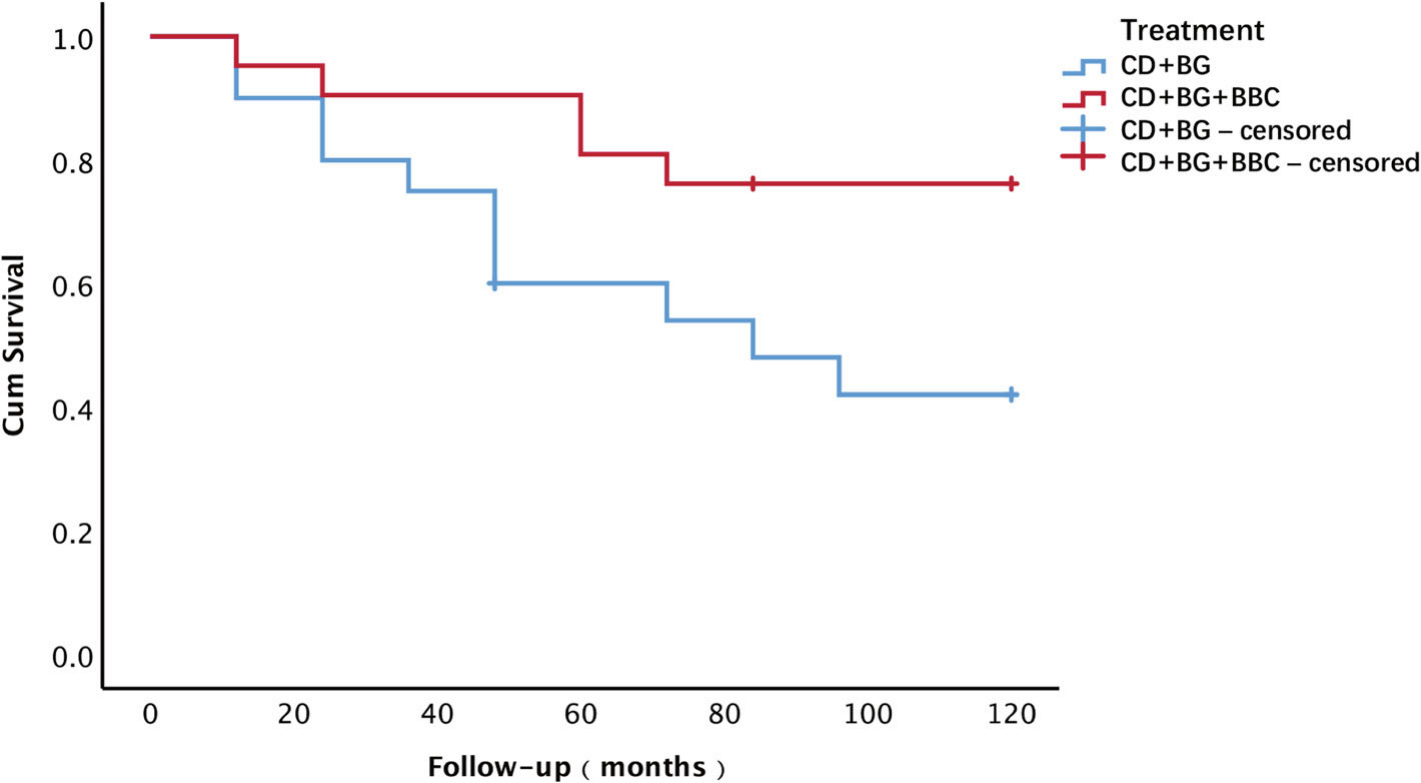
Kaplan-Meier survival curve showing femoral head survival dependent on the treatment. CD core decompression, BG bone grafting, BBC bone marrow buffy coat

The CD + BG + BBC group showed a clinical failure rate of 14.3% (3/21) at the 5-year follow-up, and the rate in the CD + BG group was 40.0% (8/20). Fisher’s exact *P* value was 0.065. At the final follow-up, the clinical failure rate was 23.8% (5/21) in the CD + BG + BBC group and 50.0% (10/20) in the CD + BG group with a Fisher’s exact *P* value of 0.078 (Fig. 3). There was a significant difference between the two groups with respect to the addition of BBC (log-rank test, *P* = 0.029) using Kaplan-Meier survival analysis (Fig. 4). The average survival times were 102.3 months and 78.1 months in the CD + BG + BBC group and CD + BG group, respectively.

**Fig. 5:**
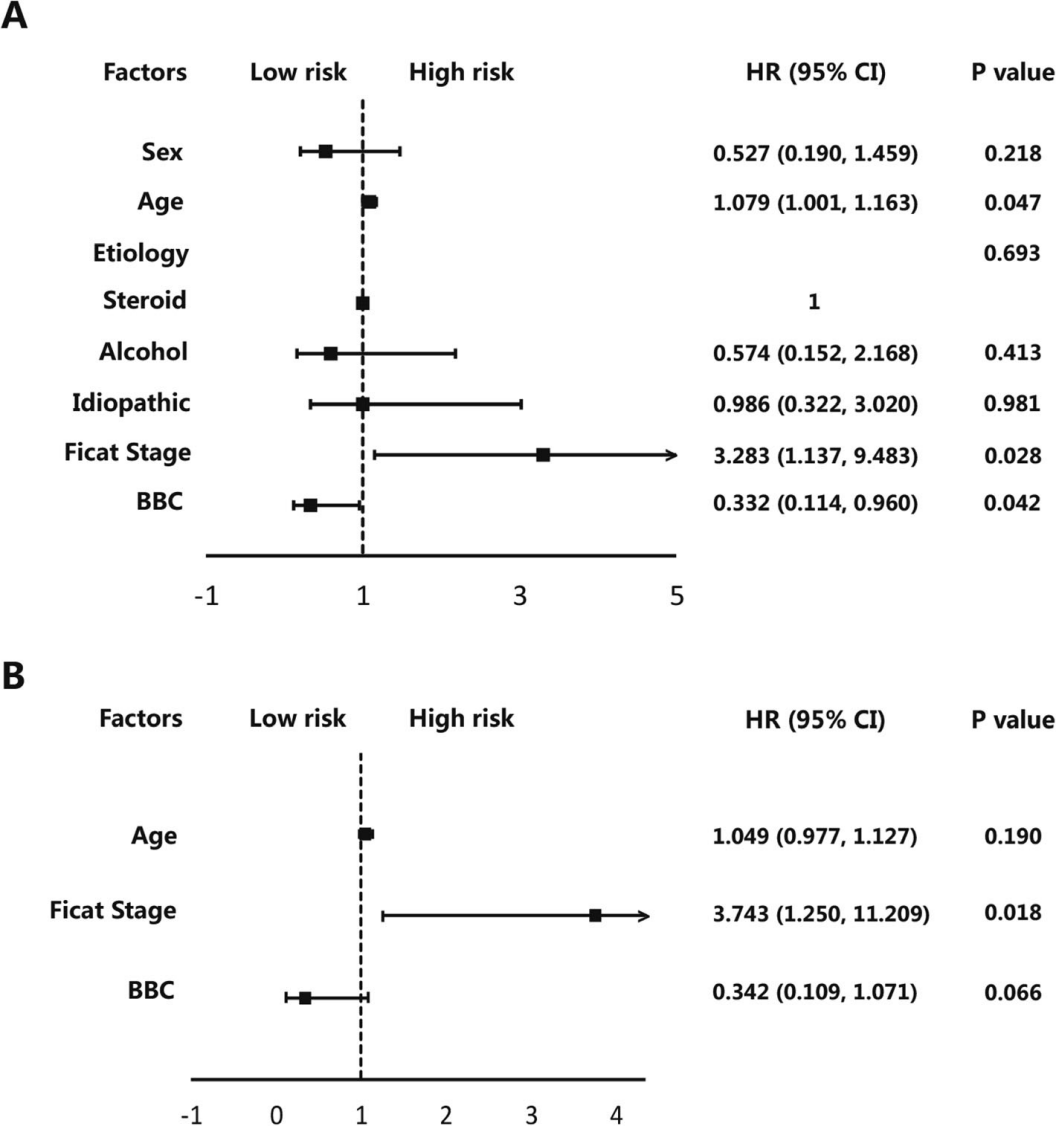
Univariate (**a**) and multivariate (**b**) Cox proportional hazards regression analysis. The forest plot of hazard ratio showed the proportional risk. BBC bone marrow buffy coat, HR hazard ratio, CI confidence interval

Univariate Cox regression analysis (Fig. 5a) showed that age [hazards ratio (HR) = 1.079, *P* = 0.047], preoperative Ficat stage (HR = 3.283, *P* = 0.028), and the addition of BBC (HR = 0.332, *P* = 0.042) were significantly associated with survivorship in a total of 41 hips. After including the aforementioned three factors in the multivariate Cox regression analysis (Fig. 5b), we found that only the Ficat stage of ANFH showed a significant association with survivorship (HR = 3.743, *P* = 0.018). Preoperative Ficat stage III hips increased the risk of progression 2.743-fold compared with Ficat stage II hips.

## Discussion

Since its initial proposal by Hernigou et al. [15], the injection of bone marrow aspirate concentrate through core decompression tunnels has been employed in more than 20 clinical studies [16]. Although the current literature includes level I and level II evidence, the follow-up period range of 2 to 5 years is relatively short. The current prospective, randomized, controlled study supported the hypothesis that the surgical procedure of combined CD, autologous bone grafting, and BBC would lead to a significantly higher survival rate on a long-term basis. This was consistent with Hernigou’s previously published result that 72% (80/114) of hips after bone marrow grafting survived at an average follow-up of 25 years, while 76% (95/125) of hips treated with single core decompression collapsed [15]. The utilization of BBC also improved the functional outcomes compared with the use of CD plus bone grafting, which is in accordance with the latest systemic review and meta-analysis [5–7].

CD, which works by drilling single or multiple tunnels from the greater trochanter through the femoral neck to the subchondral lesion of the femoral head, is the most commonly performed minimally invasive procedure for pre-collapse ANFH. It is potentially beneficial for relieving the increased bone pressure and thus promotes healing of the femoral head [17]. However, CD might deprive subchondral mechanical support as well as the quantity and quality of regional MSCs, thereby leading to insufficient bone remodelling and angiogenesis. This corresponds to the result that the clinical failure rate of single CD reaches as high as 30%, even for Ficat stage I/II ANFH hips [4, 17]. In the present study, we used the healthy part of autologous cancellous bone that was obtained from the greater trochanter and femoral neck. Furthermore, bone graft impaction through the CD track was applied to enhance the subchondral mechanical support. Although this technique showed diverging results on a long-term basis [1], we consider bone graft impaction technically easy to conduct with no risk of additional complications.

MSCs are desirable in the treatment of ANFH because of their capacity for multipotent differentiation. The purpose of adding MSCs into the core decompression tunnel is to provide osteoprogenitor and vascular progenitor cells in the area of decompressed necrotic bone to facilitate tissue regeneration and repair [18]. Among the various types of MSCs, bone-marrow-derived MSCs (BM-MSCs) are most commonly used because of their superiority in bone and cartilage repair. Additionally, BM-MSCs can release exosomes that contain cytokines promoting osteogenesis, chondrogenesis, and angiogenesis, including bone morphogenetic protein-2, vascular endothelial growth factor, and transforming growth factor-beta [4, 19, 20]. As was used in other published studies, we utilized BBC as the source of BM-MSCs, which was technically easy to harvest, and there was no need for ex vivo culture. In addition to BM-MSCs, BBC contains endothelial progenitors and haemangioblasts that contribute to vessel reconstruction and thus improve the avascular microenvironment. Based on the current literature [5, 15], the reasonable cell number for treatment ranges from 10^6^ to 10^9^, so BBC containing 10^9^ nucleated cells in the present cohort was suitable in this cell therapy.

In the present study, we detected prognostic factors using the Cox proportional hazards regression model. In the univariate model, age and preoperative Ficat stage indicated a high risk for progression, while the use of BBC indicated a low risk. Nonetheless, only preoperative Ficat stage was isolated as an independent risk factor in the multivariate model. This result is in line with the current literature that collapse-stage lesions show a worse survival rate compared with pre-collapse-stage lesions [9, 11–13, 21, 22]. This may be attributed to the decreased replication and increased apoptosis rate of osteoblasts and osteocytes in Ficat stage III femoral head lesions [17]. From a molecular point of view, Ying et al. [23] reported a high inflammatory cytokine level in the femoral head, while significant microfracture was detected. Therefore, a larger number of MSCs may be necessary to treat advanced lesions. Age-related atrophy of MSCs has been reported as a cause of decreased number and capacity of differentiation of MSCs, leading to decreased bone formation [24]. However, the hazard ratio of age was not significant in our multivariate model. Additionally, steroid use and alcoholism have been suggested to influence the treatment outcome because MSCs in these patients not only have impaired activity but also tend to differentiate into adipose cells rather than osteoblasts [4, 25]. Therefore, this aetiology was considered a prognostic factor for hip preservation, but it failed to be isolated in our model. However, due to the limited number of patients in each subclass of aetiology, we were not able to conduct an analysis of different subclasses, so future studies with larger sample sizes may provide insights.

This study had some limitations. First, as mentioned above, the number of patients included in the final analysis was small, so we did not perform subgroup analysis with respect to aetiology. Second, we included patients with bilateral ANFH, so the potentially similar performance of the bilateral hips may have led to a contralateral effect related to cell therapy. Third, we did not obtain MRI images postoperatively, which interfered with the accuracy of staging. Last but not least, there was a lack of evidence of the fate and track of the implanted cells, so the use of a cell labelling technique would be beneficial.

## Conclusion

The 10-year follow-up results of this prospective, double-blinded, randomized, controlled study showed that the use of autologous BBC in combination with core decompression was more effective than the use of core decompression alone. The preoperative Ficat stage was an independent risk factor for predicting the postoperative survival rate. Ficat stage III hips had a higher risk for progression.

## Data Availability

The data and materials used and/or analysed during the current study are not publicly available but available from the corresponding author on reasonable request.

## Abbreviations

ANFH: Avascular femoral necrosis of femoral head
THA: Total hip arthroplasty
MSC: Mesenchymal stem cell
BBC: Bone marrow buffy coat
CD: Core decompression
BG: Bone grafting
HR: Hazard ratio
VAS: Visual analogue scale
WOMAC: Western Ontario and McMaster Universities Arthritis Index osteoarthritis scoring
